# A further insight into the sialome of the tropical bont tick, *Amblyomma variegatum*

**DOI:** 10.1186/1471-2164-12-136

**Published:** 2011-03-01

**Authors:** José MC Ribeiro, Jennifer M Anderson, Nicholas C Manoukis, Zhaojing Meng, Ivo MB Francischetti

**Affiliations:** 1 Laboratory of Malaria and Vector Research, National Institute of Allergy and Infectious Diseases, National Institutes of Health, Rockville, MD 20892, USA; 2 US Pacific Basin Agricultural Research Center, Agricultural Research Service, United States Department of Agriculture, 64 Nowelo St., Hilo HI, USA; 3 Laboratory of Proteomics and Analytical Technologies, SAIC-Frederick, Inc., National Cancer Institute at Frederick, National Institutes of Health, Frederick, MD, USA

## Abstract

**Background:**

Ticks--vectors of medical and veterinary importance--are themselves also significant pests. Tick salivary proteins are the result of adaptation to blood feeding and contain inhibitors of blood clotting, platelet aggregation, and angiogenesis, as well as vasodilators and immunomodulators. A previous analysis of the sialotranscriptome (from the Greek *sialo*, saliva) of *Amblyomma variegatum *is revisited in light of recent advances in tick sialomes and provides a database to perform a proteomic study.

**Results:**

The clusterized data set has been expertly curated in light of recent reviews on tick salivary proteins, identifying many new families of tick-exclusive proteins. A proteome study using salivary gland homogenates identified 19 putative secreted proteins within a total of 211 matches.

**Conclusions:**

The annotated sialome of *A. variegatum *allows its comparison to other tick sialomes, helping to consolidate an emerging pattern in the salivary composition of metastriate ticks; novel protein families were also identified. Because most of these proteins have no known function, the task of functional analysis of these proteins and the discovery of novel pharmacologically active compounds becomes possible.

## Background

The tropical bont tick, *Amblyomma variegatum*, is a major pest of ruminants in Africa [1-3], causing skin lesions [4] and most importantly by vectoring the obligate intracellular proteobacterium *Erlichia ruminatium*, the causative agent of heartwater or cowdriosis in ruminants [5]. Although originally from Africa, *A. variegatum *has been established in the West Indies and is an important threat to domestic ruminants in the Americas [5,6].

Among the adaptations found in ticks for successful blood feeding, their salivary glands (SGs) have compounds that counteract host hemostasis and inflammation, including anticlotting, antiplatelet, vasodilatory, antihistaminic, antileukotriene, anticomplement, antichemokine, and immune-modulatory compounds [7-11]. During the past 10 years, the peptidic composition of tick saliva has been inferred from transcriptome studies, where hundreds of polypeptides are associated with a salivary function in at least 25 broad groups of protein families [7,12]. Perhaps because secreted salivary proteins are under attack by host antibodies, their rate of evolution is fast; conceivably, it is for this reason that there are many salivary protein families that are, at the primary sequence level, unique to the organism's genus level. Tick salivary compounds are of interest for providing insight into the evolution of blood feeding by arthropods, for their possible use as vaccine targets to suppress ticks or the diseases they transmit, and for presenting a platform of novel pharmacologically active compounds.

Eight years ago, a pioneer salivary transcriptome analysis of the metastriate tick *A. variegatum *was performed following the sequencing of near 4,000 salivary cDNA clones from blood-feeding adult ticks [13]. In the same year, transcriptome analysis of *Amblyomma americanum *and *Dermacentor variabilis *[14] as well as of the prostriate tick *Ixodes scapularis *[15] were performed. These three papers represent a landmark in tick biology by providing insights into their salivary composition. In these last 8 years, there was progress in the number of sialotranscriptomes (from the Greek *sialo*, saliva) sequenced, including representative species of the soft ticks, as well, as in the depth of their analysis. Many unique tick families were thus identified and reviewed [7,16]. We recently had the opportunity to collect *A. variegatum *from cows in the cattle market of Kati, Mali, a suburb of the capital city, Bamako. We separated the SG homogenate by gel chromatography and performed tryptic digest of protein bands, followed by mass spectroscopy (MS) analysis of these fragments. We re-analyzed data from Nene et al. [15], available at DBEST http://www.ncbi.nlm.nih.gov/nucest of the National Center for Biotechnology Information (NCBI), producing an annotated and hyperlinked spreadsheet containing new information related to unique tick proteins unavailable in 2002. This database was used in conjunction with proteomic analysis to identify expressed peptides. We also submitted over 600 coding (protein) sequences to GenBank, making these invaluable data available in their non-redundant (NR) database, which has only five sequences from *A. variegatum *as of June, 2010. Nucleotide sequence data reported are available in the Third Party Annotation Section of the DDBJ/EMBL/GenBank databases under the accession numbers TPA: BK007105-BK007849.

## Results and Discussion

### cDNA library characteristics

A total of 3,985 clones from the original SG cDNA library of *A. variegatum *was assembled using a combination BLAST and CAP3 pipeline [17], producing 2,077 NR sequences, or unigenes, 1,588 of which are singlets; the remaining contigs were assembled from 2 to 161 expressed tag sequences (ESTs). This assembly compares well with the TIGR assembly [13], which generated 2,109 unigenes with 1,631 singlets.

Based on various BLAST sequence comparisons to several databases (see Methods and Additional file 1), these unigenes were functionally characterized into the following groups: Putative secreted (S), putative housekeeping (H), transposable element-derived (TE), and of unknown class (U), because they could not be classified (some of which may derive from untranslated regions of mRNAs) (Table 1). Thirty-two percent of ESTs belonged to the S class, smaller than other sialomes of insects and prostriate ticks (which are ~50% of the total ESTs) but similar to metastriate sialomes of *Rhipicephalus sanguineus*, which had only 26% of its salivary ESTs attributed to the S class [18]. The S class was further subdivided in groups according to a previous tick salivary classification [7].

**Table 1:** Functional characterization of the sialotranscriptome of *Amblyomma variegatum*

Class	Total ESTs	Total contigs	EST/Contig
**Putative secreted**			
Group 1 Glycine rich superfamily	749	56	13.4
Group 2 - Mucins	24	7	3.4
Group 3 - Antigen 5 proteins	2	2	1.0
Group 4 - Ixodegrin superfamily	1	1	1.0
Group 6 - Protease Inhibitor domains			
*Group 6.1 - Kunitz domain containing proteins*	25	11	2.3
*Group 6.2 - Serpins*	6	5	1.2
*Group 6.3 - Cystatin*	1	1	1.0
*Group 6.4 - Thyropin family*	2	1	2.0
*Group 6.5 - TIL domain containing proteins*	4	2	2.0
*Group 6.6 - Hirudin/Madanin/Variegin superfamily*	5	3	1.7
*Group 6.7 - Basic tail superfamily*	3	3	1.0
Group 7 - Lipocalins	13	9	1.4
Group 8 - 8.9 kDa polypeptide family	3	2	1.5
Group 11 - 12 kDa family	4	3	1.3
Group 16 - Enzymes	115	63	1.8
Group 17 - Immunity related	7	6	1.2
Group 18 - Metastriate specific families	266	184	1.4
Group 21 - Secreted conserved proteins	60	20	3.0
			
**Group 22 - Possible housekeeping proteins**			
Unknown conserved	280	199	1.4
Protein synthesis machinery	210	73	2.9
Protein modification machinery	206	95	2.2
Metabolism, energy	196	72	2.7
Signal transduction	189	127	1.5
Transcription machinery	130	87	1.5
Protein export machinery	127	90	1.4
Transporters	115	57	2.0
Cytoskeletal Proteins	114	50	2.3
Metabolism, carbohydrate	104	50	2.1
Nuclear regulation	101	54	1.9
Transcription factor	72	50	1.4
Proteasome machinery	65	51	1.3
4Metabolism, amino acid	64	38	1.7
Metabolism, lipid	50	30	1.7
Detoxification	33	27	1.2
Metabolism, nucleotide	30	18	1.7
Extracellular matrix and adhesion	29	13	2.2
Metabolism, intermediary	28	22	1.3
Nuclear export machinery	7	6	1.2
			
**Transposable elements**	24	23	1.0
**Unknown**	521	466	1.1
			
**Total**	3985	2077	

The H class was further characterized (again based on similarities to various databases, in particular the KOG and Gene Ontology [GO] databases) into 20 functional groups (Table 1), the unknown conserved class being the most prevalent [19].

Transposable element (TE) sequences are commonly found in sialotranscriptome. The sialotranscriptome of *A. variegatum *revealed both TE class I and class II transcripts, including Tigger/Pogo transposases. These sequences may represent active transposition or, more likely, the expression of regulatory sequences that might suppress the DNA transposition phenomena [20], as indicated by a Tigger transposase message containing a stop codon (unigene amb_var-contig-1376).

### Analysis of the *A. variegatum *sialotranscriptome

Several clusters of sequences coding for H and S polypeptides (indicated in Additional file 1) are abundant and complete enough to extract consensus sequences that are typically absent from either GenBank or Swissprot. This analysis provides over 700 coding sequences, 605 of which have been submitted to GenBank through the third-party annotation system. It is to be noted that as of July, 2010, there were only five protein sequences for *A. variegatum *deposited in GenBank. These extracted sequences were grouped together in Additional file 2. A detailed description of the sialotranscriptome of *A. variegatum *follows to serve as a guide to browsing the two additional files. These two files are crosslinked to the TIGR GeneID assembly and annotation.

### Possibly secreted (S) class of expressed genes

This analysis is organized according to the groups of proteins indicated in our previous review [7].

#### Group 1: Glycine-rich superfamily

This group of proteins represent the largest group of salivary ESTs from *A. variegatum *(Table 1 and Additional file 1), totalling 749 ESTs and 56 unigenes from which 44 coding sequences (CDS) were extracted (Additional file 2). The saliva of metastriate ticks is rich in glycine-rich proteins--many of which resemble spider filaments and mostly probably function in tick attachment to their hosts--and have been targets of anti-tick vaccines [21-24]. This group also includes smaller peptides, some of which are rich in glycine and tyrosine and resemble nematode antimicrobial peptides [25].

#### Group 2: Mucins

Under this class we include diverse serine + threonine-rich secreted proteins that have in common a large number of potential O-N-acetylgalactosylation sites as identified by the NetOGlyc server [26] and can thus can be categorized as mucins. Such proteins have been regularly found in sialotranscriptomes of insects and ticks, where they are postulated to help maintain the insect mouthparts in addition to other possible functions. Ten such proteins are described in Additional file 2, including members with a chitin-binding domain.

#### Group 3: Antigen 5 proteins

The CAP superfamily of proteins (comprising the CRISP, Antigen-5, and pathogen-related-1 families) has been found in most sialotranscriptomes of insects and ticks studied to date in the form of proteins similar to wasp-venom proteins and annotated as antigen-5 [27]. The functions of these proteins are very diverse, being associated with toxins in snake venoms, [28], proteolytic activity in snails [29], and immunoglobulin binding in salivary proteins of the stable fly [30]. For example, a member of this family expressed in tabanid SGs contains a disintegrin (RGD) domain and functions as a platelet aggregation inhibitor [31,32]. To date, no tick salivary members of this family have been functionally characterized. A 3' truncated CDS for a member of this family is shown in Additional file 2.

#### Group 4: Ixodegrin superfamily

Members of this family have 110-120 amino acids (aa), many of which have the disintegrin Arg-Gly-Asp (RGD) domain with nearby cysteine residues, a motif associated with disruption of fibrinogen binding to platelets [33]. The *A. variegatum *protein named Amb_var-991 has similarities to *I. scapularis *ixodegrins, but it does not have the RGD domain. Amb_var-991 is also similar to proteins annotated as astakine, which are related to the growth factor prokineticin, which is important for hematopoiesis [34,35].

#### Group 6: Protease-inhibitor domains

##### Kunitz domain-containing proteins

The Kunitz domain is associated with proteins containing serine protease inhibitor activity as well as channel blockers. A single Kunitz domain protein from *R. appendiculatus *was identified as a potassium channel blocker, [36] while dual and five Kunitz domain proteins from *I. scapularis *were identified as clotting inhibitors by acting on the tissue factor pathway [37,38]. Additional file 2 presents 11 CDS for Kunitz domain-containing proteins from *A. variegatum *including Amb_var-163, with four Kunitz domains, and Amb_var-1788, Amb_var-68, Amb_var-995, and Amb_var-69 with three domains, as indicated by the KU Smart motif.

##### Serpins

Serpins are a ubiquitous protein family associated with the function of serine protease inhibition, from which the family name derives. *A. variegatum *serpins were identified in the original 2002 sialome publication. Four truncated CDS are presented in Additional file 2. A single tick salivary serpin from *I*. ricinus has been shown to inhibit vertebrate elastase and to have immunosuppressive activity [39,40]. Another salivary serpin from the same tick inhibits cathepsin G and chymase [41]. Tick serpins have been proposed as anti-tick salivary vaccines, including non-salivary expressed serpins [42,43].

##### Cystatins

Cystatins are cysteinyl protease inhibitors of nearly 100 aa in length. Two salivary cystatins from *I. scapularis *have been functionally characterized as inhibitors of cathepsins L and S, to inhibit inflammation, suppress dendritic cell maturation, and serve as vaccine targets [44-46]. A 3' truncated member of this family is available in Additional file 2.

##### Thyropins

Thyropins are motifs found in thyroglobulins and in cysteine protease inhibitors of the actiniam-derived equistatin protein [47-49]. Equistatin itself has three thyropin domains, two of which were shown to be involved in protease inhibition[49]. Two thyropin domains are discernible in Amb_var-355 (Additional file 2). No functional analysis of any tick thyropin has been done to date.

##### Trypsin inhibitor-like (TIL) domain-containing proteins

The TIL domain is found in some serine protease inhibitors and antimicrobials [50]. Peptides of this family have been isolated from tick eggs and shown to be inhibitors of elastase and subtilisin and to have antifungal activity [51]. The CDS of Amb_var-204 represents a salivary member of this family found in *A. variegatum*.

##### Hirudin/Madanin/Variegin superfamily

This is a superfamily found only in metastriate ticks [7] and includes the previously described peptide variegin from *A. variegatum*, shown to have antithrombin activity [52]; it also contains madanin, an antithrombin from the tick *Haemaphysalis longicornis *[53,54], and a related protein from *A. variegatum *deposited in GenBank in 2004 (accession number BAD29729.1). Additional file 2 presents three additional members of this hirudin-like protein family, characterizing its possible multigene status within *A. variegatum*.

##### Basic tail and 18.3-kDa superfamily

The basic tail and 18.3-kDa superfamily was first recognized in *I. scapularis*, where many members have repeats of basic aa in their carboxytermini. Other members have an acidic tail, and others lack the charged tail but can be recognized by the PFAM domain named tick salivary peptide group 1. The *I. scapularis *18.3-kDa family was found by PSI-BLAST to be related to the basic tail family. Two members of this family in *I. scapularis *have been characterized as anticlotting agents [55,56]. Additional file 2 introduces the CDS for four members of this family from *A. variegatum*.

#### Group 7: Lipocalins

Lipocalins are ubiquitous proteins characterized by a barrel shape that often carries lipophylic compounds (lipocalin literally means lipid cup). In blood-sucking insects and ticks, lipocalins bind not only lipidic compounds, such as leukotrienes and thromboxane A_2 _[57-59], but also charged agonists of inflammation, such as serotonin and histamine [57,60,61]. Lipocalins can also have functions unrelated to their small molecule binding function, such as anticlotting [62] and anticomplement function [63]. Seven CDS for *A. variegatum *lipocalins are presented in Additional file 2.

#### Group 8: 8.9-kDa polypeptide family

The 8.9-kDa polypeptide family is exclusive to hard ticks, 60 members of which were described previously [7]. Amb_var-1080 represents an *A. variegatum *member of the family.

#### Group 11: 12-kDa polypeptide family

**Figure 1:**
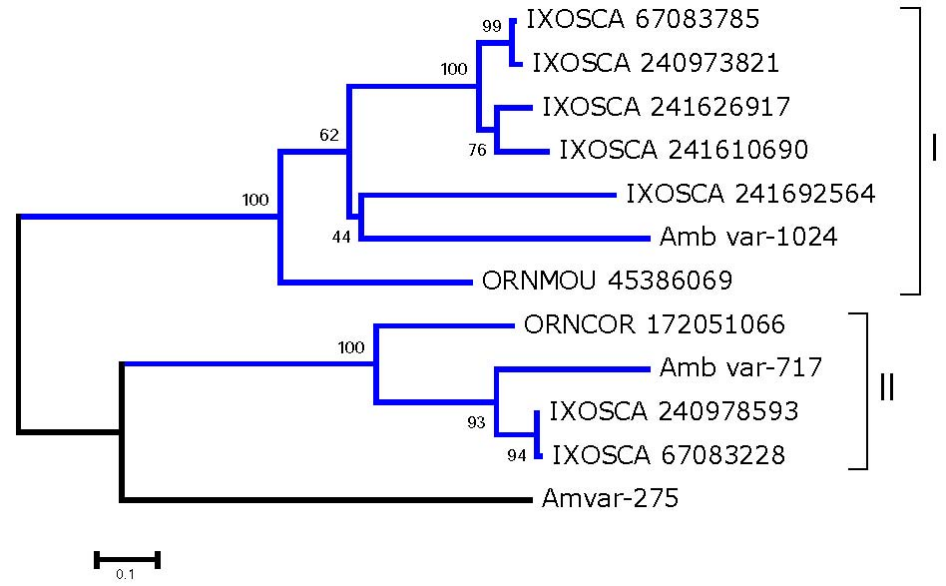
**Bootstrapped phylogram of the 12 kDa family of tick proteins**. *Amblyomma variegatum *proteins are recognized by their Amb_var or Ambvar prefix. The remaining sequences were obtained from GenBank and have six letters (three from the genus and three from the species name) followed by their NCBI accession number. The numbers near the branches indicate the percentage bootstrap support. The bar at the bottom indicates 10% amino acid divergence.

This is another protein family exclusive of hard and soft ticks, previously found only in *I. scapularis*, *Ornithodoros coriaceus*, and *Ornithodoros. moubata *[7]. Three members from *A. variegatum *are shown in Additional file 2, expanding the members of this unique family. Alignment of the tick sequences allows for a bootstrapped phylogram indicating strong bootstrap support for two clades (Figure 1).

#### Group 14: Cytotoxin-like family

Thus far, the cytotoxin-like protein family has been found only in the *Ixodes *genus and in soft ticks. Two additional proteins from *A. variegatum *add metastriate proteins to this unique tick family.

#### Group 16: Enzymes

Some of the enzymes listed below could serve an H function, but are related to enzymes previously found secreted and thus are described in the S group.

#### Apyrase/5' nucleotidases

Apyrases are enzymes that hydrolyze tri- and di-phosphonucleotides to their monophosphate esters plus inorganic phosphate. They are commonly found in the saliva of blood-sucking arthropods, where they degrade ATP and ADP, important agonists of neutrophil [64,65] and platelet aggregation [66]. The salivary apyrase of mosquitoes, triatomines of the genus *Triatoma *and ticks have been identified as members of the 5'-nucleotidase family [67-71]. While most members of the 5' nucleotidase family are membrane-bound ectoenzymes by virtue of a glycosylinositol lipid anchor, the secreted apyrases lack the carboxyterminus region where the anchor is located. Amb_var-450 (Additional file 2) is a 3' truncated member of this family, and for this reason, the lack of the anchor site cannot be evaluated.

#### Endonucleases

Endonucleases were found in saliva of *Culex *and sand flies, where they may serve a function in decreasing the viscosity of the feeding lesion and produce antiinflammatory nucleotides [72-74]. Three truncated members of this family of enzymes are presented in Additional file 2.

#### Sphingomyelin phosphodiesterases

Sphingomyelin metabolites are important regulators of cell growth, inflammation, and immunity [75,76]. A fragment of an enzyme-targeting sphingomyelin is identified.

#### Epoxide hydrolases

Two truncated members of this family of enzymes were found that could act in the metabolism of arachidonate metabolites.

#### Oxidant metabolism enzymes

A peroxidasin fragment, a superoxide dismutase, and two selenoproteins are reported in Additional file 2. These proteins have the potential to regulate the toxic products of oxygen and nitric oxide [77,78].

#### Proteases

Carboxypeptidases, dipeptidyl peptidases, metalloproteases of the reprolysin family, and trypsin-like serine proteases are presented in Additional file 2. Carboxypeptidases and dipeptidyl peptidases could act in the destruction of inflammatory peptidic agonists. In fact, a dipeptidyl peptidase was shown to be responsible for the very fast bradykinin degradation caused by *I. scapularis *saliva [79]. Metalloproteases in the saliva of *I. scapularis *were shown to be responsible for the fibri(noge)nolytic activity [80]. Salivary serine proteases have been shown to have fibrinolytic activities in horse flies [31].

#### Group 17: Immunity related

A member of the ficolin family, named ixoderin in ticks [81], is identified. These proteins have a lectin and a fibrinogen-like domain and are associated with activation of the colectin pathway of complement activation in vertebrates and invertebrates [82].

#### Group 18: Metastriate-specific families

Several protein families found only in metastriate ticks were identified in our previous review of tick sialomes [7]. Seven of these families were also found in the *A. variegatum *sialome, including three multigene families that appear to be unique to *A. variegatum*, as follows:

##### Da-p36 immunosuppressant

**Figure 2:**
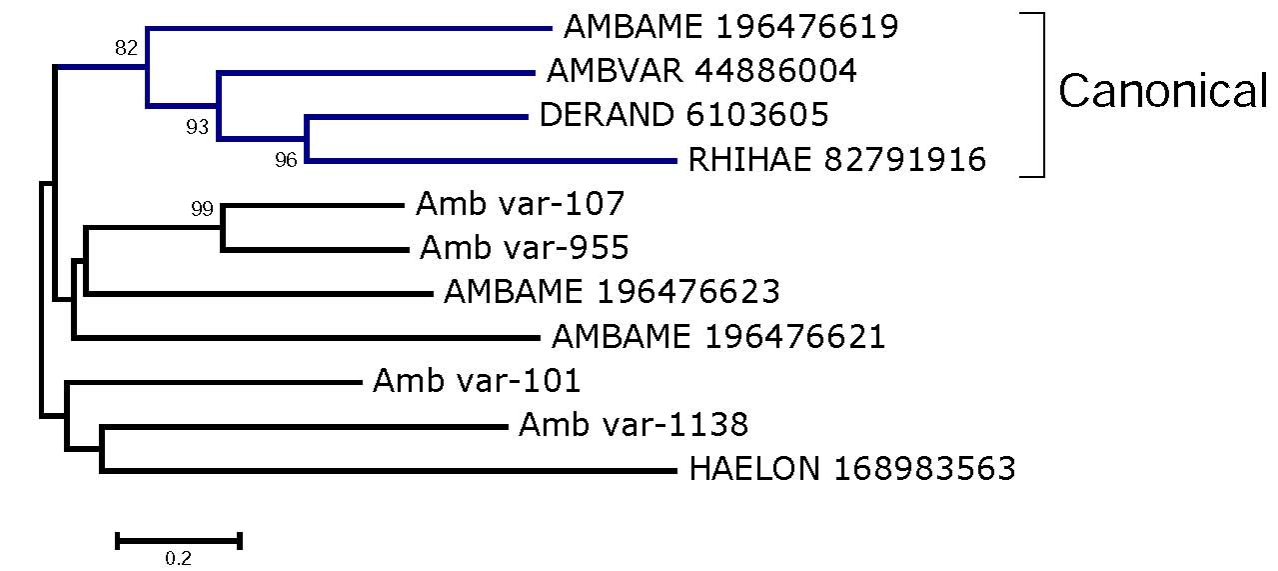
**Bootstrapped phylogram of the Da-p36 kDa family of metastriate tick proteins**. *Amblyomma variegatum *proteins are recognized by their Amb_var or AMBVAR prefix. The remaining sequences were obtained from GenBank and have six letters (three from the genus and three from the species name) followed by their NCBI accession number. The numbers near the branches indicate the percentage bootstrap support (values below 50% are not shown). The bar at the bottom indicates 20% amino acid divergence. Bootstrap was performed with 1000 iterations.

The first member of this family was described as an immunosuppressant found in the SGs of *Dermacentor andersoni *[83]. The *A. variegatum *sialome reveals four members of this family, one of which was reported in Figure 2 of the original 2002 paper [13]. One additional sequence from *A. variegatum *was deposited in GenBank in 2004 (accession number BAD11807.1), but has no associated publication. Alignment of the related sequences (not shown) reveals one small conserved region characterized by the block [ILF]-x(3)-[IMLF]-x-[SCA]-P-[FM]-x(4)-[NT]-[VLIFM]-x-[ILFV], indicating the divergence of the family. The bootstrapped phylogram shows strong support for one clade containing *Amblyomma*, *Dermacentor*, and *Rhipicephalus *sequences (Figure 2). Other clades do not have sufficient bootstrap support (>50%), except for two *A. variegatum *sequences that are over 20% divergent. The phylogram supports a common origin of this multigene family in metastriate ticks.

##### Metastriate insulin growth factor-binding protein

**Figure 3:**
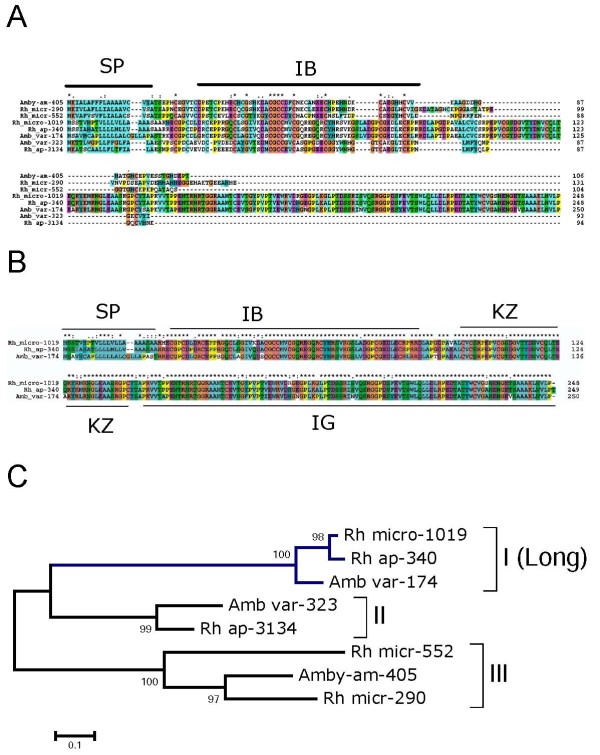
**Tick metastriate proteins containing the SMART insulin growth factor binding (IB) domain**. (A) All proteins. (B) Proteins containing IB domain plus immunoglobulin (IG) and Kazal (KZ) domains. SP indicates the signal peptide region. (C) Bootstrapped phylogram of the alignment in (A). The symbols above the alignment indicate (*) identity, (:) similarity, and (.) less conserved similarity.

This protein family was discovered from the assembly of ESTs from ticks, as described in a previous review [7]. Members of this family produce matches to GO proteins annotated as "insulin-like growth factor binding protein 7" and have the IB SMART domain for "insulin growth factor-binding protein homologues." This family actually has two sets. A shorter form contains only the IB domain (Figure 3A), while the longer form has, in addition of the IB domain, a Kazal domain and the SMART immunoglobulin C-2 type domain (Figure 3B). Alignment of all IB-containing proteins (Figure 3A) shows the conserved IB domain motif in the amino terminal end of the mature protein, indicated by the block PA C-x(12,13)-[EQD]-C-x(2)-G-x(5)-C-G-C-C-x(2)-C-x(5)-[EQD]-x-PA C-x(7,13)-C-x-[EK]-x(3)-C. Alignment of the long-form sequences shows three very conserved proteins in *Rhipicephalus microplus*, *R. appendiculatus*, and *A. variegatum*, containing a signal peptide followed by the IB, Kazal, and immunoglobulin domains (Figure 3B). This degree of conservation is more common among housekeeping proteins, as they are not under host immune pressure. Proteins containing these three domains are characterized by the InterPro insulin-like growth factor binding protein 7 http://www.ebi.ac.uk/interpro/ISpy?ac=Q16270. The human homolog having this domain structure (GenBank accession number NP_001544.1), named MC25 or IGFBP7, has many effects in tissue growth and differentiation [84,85], It has also been shown to inhibit vascular endothelial growth factor and keratinocyte growth [86,87]. If any of these proteins are secreted, they could serve as binders of growth factors affecting angiogenesis, tissue repair, and immunity.

##### *A. variegatum*-specific proteins

Additional file 2 contains two families of proteins that appear to be species specific, namely the Avar family10 kDa (three genes) and Avar family 8 kDa (two genes). Within each family, the members are less than 50% identical, indicating gene duplication events followed by divergence. Additional file 2 presents 79 additional protein sequences that have a putative signal peptide indicative of secretion but have no similarity to any known protein, including the recently released *I. scapularis *proteome. It is possible that some of these CDS may derive from the 3' region of transporters or other transmembrane proteins, as these regions may produce a positive signal peptide.

##### *A. variegatum *proteins not found in previous sialotranscriptome, but similar to putative *I. scapularis *proteins

Additional file 2 contains 13 proteins from *A. variegatum *that are similar to *I. scapularis *proteins but have not been found in previous sialomes. Some of these are proline-rich, low-complexity proteins or histidine-rich proteins.

#### Group 21: Secreted conserved proteins

Forty-five proteins with signal peptide indicative of secretion are presented in Additional file 2. Most of these are of the class "Unknown conserved" [19] but also include calreticulin, which has a typical KHEEL carboxydomain indicative of endoplasmic reticulum retention but was shown to be a marker of tick exposure [88].

#### Group 22: Housekeeping proteins

Additional file 2 presents 414 CDS for proteins associated with various cellular functions. Additionally, 40 of the unknown conserved and 3 transposable element fragments were extracted.

### Preliminary characterization of the salivary proteome of *A. variegatum*

**Figure 4:**
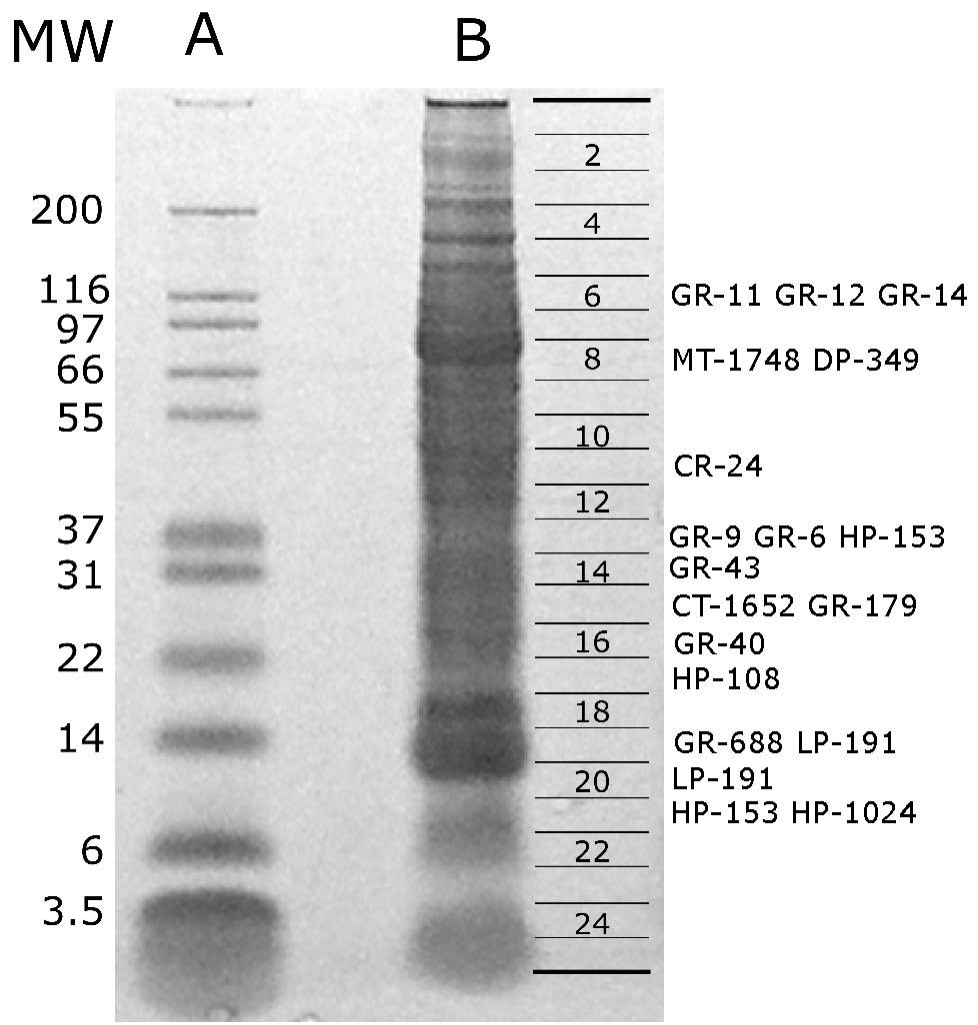
**Sodium dodecyl sulfate polyacrylamide gel electrophoresis of salivary gland homogenate of *Amblyomma variegatum***. Lane A shows molecular mass markers, with their masses indicated in column MW. Lane B represents the tick sample. The grid at the right indicates how the gel was cut for tryptic digest of the peptides, and the two-letter-number code indicates the putative secreted proteins identified at the gel bands. The two-letter codes stand for: GR, glycine-rich protein; MT, metalloprotease; DP, dipeptidyl peptidase; CR, calreticulin; HP, hypothetical protein; CT, cytotoxin-like; and LP, lipocalin. The numbers indicate the Amb_var protein number available in Supplemental Table S2.

To obtain information on protein expression in the SGs of *A. variegatum*, we performed a one dimensional (1D) gel electrophoresis separation of the SG homogenate followed by proteolytic digest of the 25 cored sections indicated in Figure 4 and subsequent tandem mass spectrometry (MS/MS) of the tryptic peptides. Additional files 1 and 2 show the matching sequence hits obtained by MS/MS. A total of 170 proteins were identified in the gel fractions by two or more ions derived from the same gel fraction. An additional 39 proteins were identified from a single ion on the same spot. These matches are shown in the worksheet named Gel-MS-MS results in Additional file 2. Figure 4 displays the electrophoresis gel, indicating 19 proteins associated with blood feeding. These include large and small glycine-rich proteins, the most abundant group found in the transcriptome and by MS. Reprolysin-type metalloprotease and dipeptidyl peptidase are found in band 8, with their expected molecular masses (just above the 66-kDa marker). Calreticulin, citotoxin-like protein, one lipocalin, and four hypothetical secreted proteins are also shown in Figure 4.

## Conclusions

The detailed re-analysis of the transcriptome of *A. variegatum*, in light of the emerging pattern of protein families in tick sialomes, extends and confirms common components in the saliva, such as the recruitment of metalloproteases, protease inhibitors, lipocalins, and several other unique families--such as the 8.9-kDa, 11-12-kDa, and cytotoxin-like--common to metastriate and prostriate ticks. *A. variegatum *also has a large set of transcripts coding for cement-like proteins unique to metastriate ticks. In parallel with this transcript abundance, glycine-rich proteins were the largest group of proteins identified by proteomics, when secreted proteins are considered. Other unique metastriate protein families were identified, including some that appear to be multigenic and also unique to *A. variegatum *such as the Avar 10-kDa and Avar 8-kDa families. Many orphan proteins were further characterized. Further transcriptome analysis of other *Amblyomma *ticks may reveal relatives of these unique proteins.

Most of the proteins described have no known function but, if secreted into their hosts, they should have antihemostatic, antiinflammatory, anti-angiogenic, or immunomodulatory function. They may also contain antimicrobial activity. As the sialome puzzle emerges, the task of functional characterization of these novel protein families becomes possible.

## Methods

### Biological material

Female *A. variegatum *ticks were obtained from zebu cattle (*Bos primigenius indicus*) at a market located in the village of Kati, located approximately 30 km north of Bamako, the capital of Mali (12°44'48.03"N, 8°04'17.09"W). The ticks were briefly washed in 70% ethanol and then air dried. The tick was secured to a glass slide using double-sided tape, and then one horizontal and two lateral cuts were made with a sterile scalpel to disconnect the SGs from the spiracles connecting them to the feeding duct and spiracular plate. The dorsal plate was then removed, exposing the midgut, SGs, and other organs. The SGs were teased away from other organs using ultra-fine forceps (#5, Bioquip) in a bath of 1 × PBS. The dissected SGs were washed in 1 × PBS before being stored in PBS. The tick carcasses were retained in 70% ethanol and submitted as voucher specimens for identification by Dmitry A. Apanaskevich, assistant curator at the United States National Tick Collection at Georgia Southern University.

### Bioinformatic tools and procedures used

ESTs from the SGs of adult female *A. variegatum *deposited in DBEST as part of a previous publication [13] were retrieved and assembled in our assembly pipeline. The BLAST tool [89] and the CAP3 assembler [90] were used to assemble the database as well as to compare it to other databases and pipe the results into a hyperlinked Excel spreadsheet, as described in the dCAS software tool [17]. ClustalW [91] and TreeView software [92] were used to align sequences and visualize alignments. Phylogenetic analysis and statistical neighbor-joining bootstrap tests of the phylogenies were done with the Mega package [93]. For functional annotation of the transcripts, we used the tool blastx [94] to compare the nucleotide sequences to the NR protein database of the NCBI and to the GO database [95]. The tool rpsblast [94] was used to search for conserved protein domains in the Pfam [96], SMART [97], Kog [98], and Conserved Domains Databases (CDD) [99]. We have also compared the transcripts with other subsets of mitochondrial and rRNA nucleotide sequences downloaded from NCBI. Segments of the three-frame translations of the EST (because the libraries were unidirectional, we did not use six-frame translations), starting with a methionine found in the first 100 predicted aa, or to the predicted protein translation in the case of complete coding sequences, were submitted to the SignalP server [100] to help identify translation products that could be secreted. O-glycosylation sites on the proteins were predicted with the program NetOGlyc [26]. Functional annotation of the transcripts was based on all the comparisons above.

When attempting identification of multigene families, we attributed transcripts coding for proteins that were more than 10% different in their primary aa sequence to derive from different genes. The reader should be aware that products divergent more than 10% could be alleles of polymorphic genes.

### Gel electrophoresis studies

Tick salivary proteins representing approximately 100 μg were resolved by one-dimensional (1D) sodium dodecylsulfate polyacrylamide gel electrophoresis (4-12% gradient gels) and visualized with Coomassie blue staining (Pierce). Excised gel bands were destained using 50% acetonitrile in 25 mM NH_4_HCO_3_, pH 8.4, and vacuum dried. Trypsin (20 μg/mL in 25 mM NH_4_HCO_3_, pH 8.4) was added and the mixture was incubated on ice for one h. The supernatant was removed and the gel bands were covered with 25 mM NH_4_HCO_3_, pH 8.4. After overnight incubation at 37°C, the tryptic peptides were extracted using 70% acetonitrile, 5% formic acid, and the peptide solution was lyophilized and desalted using ZipTips (Millipore).

### Nanoflow reverse-phase liquid chromatography tandem mass spectrometry (nanoRPLC-MS/MS)

Tryptic peptides were analyzed using nanoRPLC-MS/MS. A 75-μm i.d. × 360-μm o.d. × 10-cm long fused silica capillary column (Polymicro Technologies) was packed with 3 μμm, 300 Å pore size C-18 silica-bonded stationary RP particles (Vydac). The column was connected to an Agilent 1100 nanoLC system (Agilent Technologies) that was coupled online with a linear ion-trap mass spectrometer (LTQ; ThermoElectron). Peptides were separated using a gradient consisting of mobile phase A (0.1% formic acid in water) and B (0.1% formic acid in acetonitrile). The peptide samples were injected, and gradient elution was performed under the following conditions: 2% B at 500 nL/min for 30 min; a linear increase of 2-42% B at 250 nL/min for 110 min; 42-98% for 30 min including the first 15 min at 250 nL/min and then 15 min at 500 nL/min; 98% at 500 nL/min for 10 min. The linear ion-trap mass spectrometer was operated in a data-dependent tandem MS (MS/MS) mode in which the five most abundant peptide molecular ions in every MS scan were selected for collision-induced dissociation using a normalized collision energy of 35%. Dynamic exclusion was applied to minimize repeated selection of previously analyzed peptides. The capillary temperature and electrospray voltage were set to 160°C and 1.5 kV, respectively. Tandem MS spectra from the nanoRPLC-MS/MS analyses were searched against a protein fasta database derived from the tick transcriptome using SEQUEST operating on an 18-node Beowulf cluster. For a peptide to be considered legitimately identified, it had to achieve stringent charge state and proteolytic cleavage-dependent cross correlation (X_corr_) and a minimum correlation (ΔC_n_) score of 0.08.

MS results were mapped to the Excel spreadsheets using a homemade program. The following example illustrates the convention for interpreting the data: The hit Band7 → 6 indicates that a particular protein had six MS/MS peptide hits in gel fraction 7. Additional columns indicate the number of residues covered in aa residues and percent of total protein that was covered by the procedure.

## Abbreviations

CDS: coding sequence; EST: expressed tag sequence; GO: Gene Ontology (database); H: housekeeping class; MS: mass spectrometry; NCBI: National Center for Biotechnology Information; NR: non-redundant; S: putative secreted class; SG: salivary gland; TE: transposable elements class; U: unknown function class.

## Authors' contributions

JMA and NM collected and dissected ticks and helped with the manuscript. JMCR performed bioinformatical analysis and wrote the bulk of the manuscript. IMBF helped with tick samples, experimental design, and writing the manuscript. ZM performed electrophoresis, tryptic digestion, and mass spectrometry analysis and contributed to the manuscript. All authors read and approved the final manuscript.

## Supplementary Material

Additional file 1 **Hyperlinked Excel spreadsheet with transcriptome data http://exon.niaid.nih.gov/transcriptome/A_variegatus/Avar-S1-web.zip**. Click here for file

Additional file 2 **Hyperlinked Excel spreadsheet with coding sequence data http://exon.niaid.nih.gov/transcriptome/A_variegatus/Avar-S2-web.zip**. Click here for file
